# Exhaustive identification of steady state cycles in large stoichiometric networks

**DOI:** 10.1186/1752-0509-2-61

**Published:** 2008-07-11

**Authors:** Jeremiah Wright, Andreas Wagner

**Affiliations:** 1 Department of Biochemistry, University of Zurich, Zurich, Switzerland; 2 Swiss Institute of Bioinformatics, Lausanne, Switzerland; 3 Sante Fe Institute, Sante Fe, New Mexico, USA; 4 Department of Biology, The University of New Mexico, Albuquerque, New Mexico, USA

## Abstract

**Background:**

Identifying cyclic pathways in chemical reaction networks is important, because such cycles may indicate *in silico *violation of energy conservation, or the existence of feedback *in vivo*. Unfortunately, our ability to identify cycles in stoichiometric networks, such as signal transduction and genome-scale metabolic networks, has been hampered by the computational complexity of the methods currently used.

**Results:**

We describe a new algorithm for the identification of cycles in stoichiometric networks, and we compare its performance to two others by exhaustively identifying the cycles contained in the genome-scale metabolic networks of *H. pylori*, *M. barkeri*, *E. coli*, and *S. cerevisiae*. Our algorithm can substantially decrease both the execution time and maximum memory usage in comparison to the two previous algorithms.

**Conclusion:**

The algorithm we describe improves our ability to study large, real-world, biochemical reaction networks, although additional methodological improvements are desirable.

## Background

Flux balance analysis is becoming a well developed and frequently used theoretical tool to study the capabilities of large stoichiometric networks [1]. Flux balance analysis relies on several assumptions that essentially impose constraints on the allowable states of a network. Ideally, these constraints are derived from fundamental physical and chemical principles so that the physically realistic states of a network can be accurately identified and the unrealistic states ignored. Ensuring the conservation of mass can be achieved in a relatively straightforward manner, but it is much more challenging to incorporate the conservation of energy [2-8]. Methods that rely on the identification of steady state reaction cycles have been developed to achieve this [3-5]. Knowledge of these cycles can be used to constrain the direction or magnitude of certain fluxes to prevent the occurrence of thermodynamically inconsistent network states.

The incorporation of energetic constraints in flux balance analysis is not the only motivation to identify cycles. Cycles may also point towards important aspects of network function. For instance, it has been proposed that cycles in metabolic networks can affect the sensitivity and robustness of network function and allow for regulation of biochemical pathways [9]. In signal transduction networks, which are also stoichiometric in nature [10,11], cycles may allow for feedback. Feedback is known to be an important property of signal transduction, and it has been shown to result in a variety of complex and potentially useful biochemical behaviors [12,13].

Two algorithms have been explicitly described for the identification of steady state cycles in stoichiometric networks. Schilling, Letscher, and Palsson (SLP) described the first of these in 2000 [14]. The SLP algorithm first defines a network of *internal *and *exchange *reactions. Internal reactions are those that are actually being studied. For metabolic networks, for example, the internal reactions are those that occur in or around the cell, such as reactions involved in glycolysis, respiration, transport of metabolites across cellular membranes, etc. Exchange reactions are pseudo-reactions that are used to supply (remove) chemical species to (from) the reaction system. The flux, or rate, of each internal reaction is constrained to be positive, while the fluxes of exchange reactions may be left unconstrained. If reversible reactions are present in the network, they are broken apart into a pair of unidirectional forward-reverse reactions, each with its own flux. These reactions are used to construct a stoichiometry matrix *S *which is used to formulate the equation *Sv = 0*, where *v *is a vector of fluxes of all reactions in the network. The solutions to this equation represent the allowable steady states of the network, where *steady *refers to the fact that the concentrations of internal chemical species remain constant.

The SLP algorithm then identifies all of the *extreme pathways *of a network, which are a unique set of flux vectors whose superposition can generate all steady-state fluxes in a network that do not violate the principle of mass conservation. The extreme pathways are then categorized based on the types of active reactions they contain. We use the word *cycles *from this point forward to refer exclusively to internal cycles, that is *type III *extreme pathways, which are the extreme pathways that do not contain active exchange reactions ([14] and see Appendix). Type III extreme pathways are also elementary flux modes [15,16] and extremal currents [17]. The identification of extreme pathways is equivalent to computing the set of extreme rays of a convex cone [14], which is known to be an NP-hard problem [16,18,19]. This computational complexity limits the size of networks for which the SLP algorithm can be used [20], although numerous algorithmic improvements have been recently made in an effort to alleviate this problem [19,21-23].

Mahadevan and Schilling (MS) very briefly described the second algorithm for cycle identification in 2003 [24]. In this approach, the network and its corresponding stoichiometry matrix are defined in the same way as for the SLP algorithm. The MS algorithm, however, uses a unique property of cycles to assist with their detection. If a network does not contain exchange reactions, and the fluxes of all internal reactions are constrained to the interval [0, ∞), the only reactions in the network capable of functioning will be those that participate in cycles (see Appendix). The MS algorithm takes advantage of this fact by using linear programming to determine the maximum flux of each reaction in the network. All of the reactions with unbounded fluxes are then used to create a sub-network of the original network. Every reaction in this sub-network necessarily participates in a cycle of the original network (see Appendix). The SLP algorithm is then applied to the sub-network to identify all of its extreme pathways, which are necessarily the complete set of cycles in the original network (see Appendix). Since the sub-network supplied to the SLP algorithm is potentially smaller than the original network, the identification of cycles in larger networks may become possible.

In this paper, we describe a new algorithm, which we refer to here as the WW algorithm. The WW algorithm is an extension of the MS algorithm, and it can reduce the size of the sub-network supplied to the SLP algorithm far beyond that of the MS algorithm. We also measure and compare, for the first time, the performances of all three algorithms using five genome-scale metabolic networks.

## Results and Discussion

### The WW algorithm

Step 1. Ensure that the network does not contain exchange reactions, and constrain the flux of each internal reaction to the interval [0, ∞).

Step 2. Let *U *denote an empty set, and for each reaction *R *in the network, do the following:

Step i. By pair-wise comparison, identify all reactions in the network that are the reverse of *R*.

Step ii. For each of the reverse reactions identified in Step i, constrain its flux to be zero.

Step iii. Determine if the flux of reaction *R *can assume a value other than zero, while still satisfying all currently defined constraints. If so, add reaction *R *to the set *U*.

Step iv. For each of the reverse reactions identified in Step i, restore the constraint on its flux to [0, ∞).

Step 3. Identify the extreme pathways in the network defined by the reactions contained in the set *U*.

Step 4. By pair-wise comparison, identify each pair of internal forward-reverse reactions, *R*_*F *_and *R*_*R*_, in the stoichiometric network. A particular *R*_*F*_-*R*_*R *_pair forms a single type III extreme pathway (see Appendix).

Step 5. Combine the results of Steps 3 and 4 to obtain the set of all cycles present in the network (see Appendix).

Step 1 ensures that the only extreme pathways present in the network are of type III, since all other types of extreme pathways must contain active exchange reactions. Step 2 is used to identify all reactions that participate in cycles composed of three or more reactions. Under these circumstances, the fluxes of reactions that participate in such cycles can assume any value on the interval [0, ∞), and the fluxes of reactions that do *not *participate in such cycles must be zero. Any technique capable of making this distinction, such as linear programming, can be used during this step. The reason for this property is that if a reaction *R *only participates in cycles composed of two reactions, it must be inactive when its reverse reactions are constrained to be inactive. In Step 3, a sub-network is created that is composed of the reactions identified in Step 2, and an algorithm is applied to that network to identify the type III extreme pathways it contains. That algorithm could be the SLP algorithm, or another that achieves the same result. Step 4 is used to identify all of the extreme pathways composed of only two reactions. This could also be accomplished during Step 2.i, but it is listed separately here for the sake of clarity. The appendix contains a more explicit justification of the principle behind the WW algorithm.

There are two key differences between the WW and MS algorithms. Firstly, the MS algorithm, as it is defined, individually maximizes the flux of each reaction in a network. Flux maximization can also be performed to accomplish Step 2.iii of the WW algorithm, but other (potentially faster) techniques could be used as well. Secondly, the MS algorithm creates sub-networks composed of all of the reactions in a network that participate in cycles. The WW algorithm, however, creates sub-networks composed only of those reactions that participate in cycles composed of three or more reactions. Consequently, the WW algorithm can sometimes produce sub-networks drastically smaller than those of the MS algorithm, which is demonstrated below.

#### Performance comparisons

**Figure 1:**
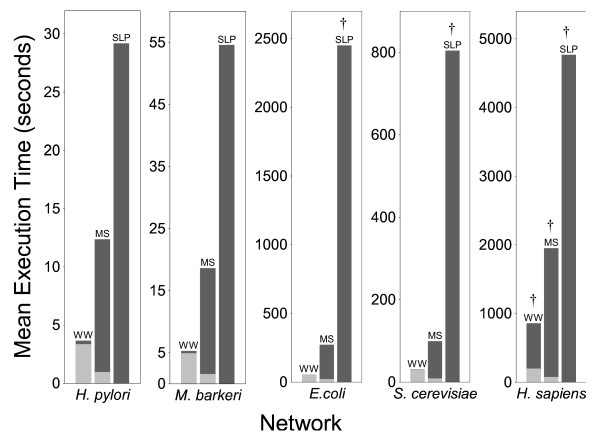
**The average execution time of each algorithm with each network.** Light grey indicates the time spent in the preprocessing phase, and dark grey indicates the time spent identifying extreme pathways. A dagger (†) directly over a bar indicates that the algorithm halted due to memory exhaustion. The indicated values are, therefore, only a lower bound on the total execution time required for successful completion.

**Figure 2:**
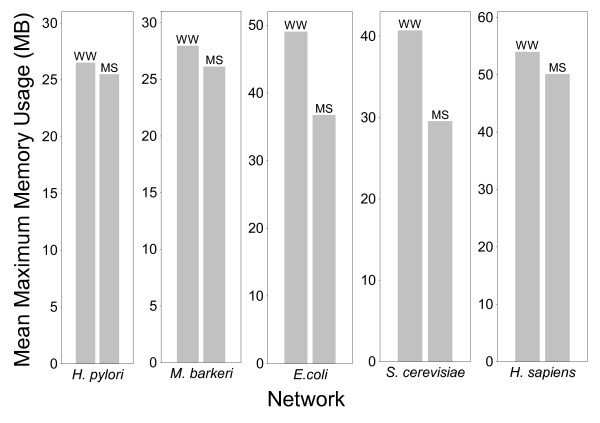
The average maximum memory consumption of the preprocessing phase of the MS and WW algorithms with each network.

**Figure 3:**
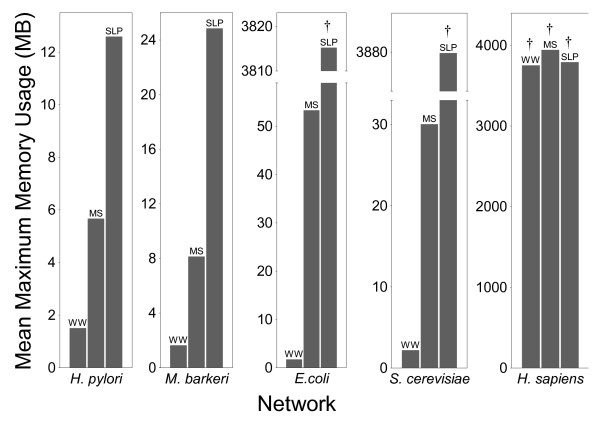
**The average maximum memory consumption of the extreme pathway identification phase of each algorithm with each network.** A dagger (†) directly over a bar indicates that the algorithm halted due to memory exhaustion. The indicated values are, therefore, only a lower bound on the maximum memory required for successful completion.

We tested the performance of the MS, SLP, and WW algorithms for genome-scale metabolic networks from four different microbes and from *Homo sapiens *(human), comprising between 486 and 2786 chemical species, and between 642 and 4482 reactions. Our results show that both execution time efficiency (Figure 1 and Additional file 1) and memory efficiency (Figures 2, 3, and Additional file 1) were substantially different for the three algorithms, with the WW algorithm being more efficient than the MS algorithm, which was more efficient than the SLP algorithm. These performance differences are described in detail in the following sections.

#### Preprocessing

Both the WW and MS algorithms use a preprocessing procedure to reduce the size of the network before identification of cycles is attempted. For both algorithms, the time and memory consumption of the preprocessing phase increases as network size increases, although not substantially; and WW preprocessing consumes more time and moderately more memory than MS preprocessing (Figures 1 and 2).

#### Extreme pathway identification

The time and memory consumption of the MS, SLP, and WW algorithms during extreme pathway identification were quite different. To begin with, the SLP algorithm was unable to complete execution with the *E. coli*, *S. cerevisiae*, and *H. sapiens *networks due to memory exhaustion. This also occurred for both the MS and WW algorithms with the *H. sapiens *network. Therefore, at least for the hardware and software configurations used for these analyses, memory efficiency during extreme pathway identification was the most important factor for achieving successful computation. For the microbial networks, the maximum memory consumption of the MS algorithm was much less than that of the SLP algorithm, and the memory consumption of the WW algorithm was less than that of the MS algorithm (Figure 3). The memory usage of both the MS and SLP algorithms increased substantially with increasing network size, but in contrast, the memory usage of the WW algorithm changed little as the size of the microbial networks increased (Figure 3). Similar trends were observed for time efficiency, as well (Figure 1).

The observed performance differences of extreme pathway identification can best be explained by noting the reduction of network sizes achieved during the preprocessing phases of the MS and WW algorithms. The SLP algorithm uses the entire network, whose numbers of reactions and metabolites are shown in Table 1. The MS algorithm uses sub-networks containing only reactions that participate in cycles, but the WW algorithm uses sub-networks containing only reactions that participate in cycles that are composed of more than two reactions. The reduction in size between the full network and the sub-network used by the WW algorithm is dramatic, spanning more than an order of magnitude for each of the microbial networks (Table 1). For these networks, the majority of cycles are composed of only two reactions (Table 2), allowing the WW algorithm to produce much smaller sub-networks than the MS algorithm. This greatly reduces the computational effort required of the SLP algorithm, resulting in better performance.

**Table 1:** Sizes of networks supplied to the SLP algorithm by each algorithm.

	**Chemical Species**			**Reactions**			**Non-Zero** **Matrix Elements**		
			
**Network**	**SLP**	**MS**	**WW**	**SLP**	**MS**	**WW**	**SLP**	**MS**	**WW**
*H. pylori*	486	251	34	642	335	26	2861	1285	110
*M. barkeri*	628	308	34	816	400	40	3781	1609	182
*E. coli*	1673	897	57	2635	1140	50	10487	3366	201
*S. cerevisiae*	1061	636	77	1580	873	98	6746	3182	384
*H. sapiens*	2786	1630	633	4482	2812	1229	17571	9677	5010

**Table 2:** Number of cycles per microbial network.

**Network**	**Cycles of Size = 2**	**Cycles of Size > 2**	**Total Cycles**
*H. pylori*	165	7	172
*M. barkeri*	200	27	227
*E. coli*	564	27	591
*S. cerevisiae*	434	39	473

#### Overall performance

Although the WW preprocessing step consumes more time and memory than MS preprocessing, the time and memory efficiency of identifying extreme pathways during the WW algorithm is dramatically better. Consequently, the overall execution time of the WW algorithm is substantially less than the MS algorithm, and the memory consumption is also significantly reduced during the phase of the computation that is most memory-intensive and most susceptible to combinatorial explosion.

#### Caveats

There are certain extreme cases where these performance differences will not persist. For example, if every reaction in a stoichiometric network participates in a cycle, the MS algorithm will fail to outperform the SLP algorithm. In this situation, the maximum flux of each reaction will be unbounded, resulting in a "sub-network" that is exactly the same as the original. Similarly, if every reaction in a stoichiometric network participates in cycles composed of more than two reactions, neither the MS nor WW algorithm will outperform the SLP algorithm. If all the cycles in a stoichiometric network are composed of more than two reactions, the WW algorithm will fail to outperform the MS algorithm. If a reaction network does not contain any cycles, the MS algorithm will likely have a performance advantage over the WW algorithm, depending on implementation details. We note that these extreme scenarios are not realistic for genome-scale metabolic networks (Table 2), the kinds of networks for which application of the WW algorithm would be most fruitful. If, finally, a network only contains cycles composed of two reactions, the WW algorithm will never make use of the SLP algorithm, which eliminates the chance of combinatorial explosion and most likely provides further dramatic performance improvements over the MS algorithm.

It should also be noted that the algorithmic performances described herein are dependent upon implementation details and the choice of underlying algorithms. Other software and algorithms [25-27], for example, could be used to identify extreme pathways, which would certainly change the time and memory requirements of these computations. Additionally, flux maximization was performed by both implementations of the WW and MS algorithms during the preprocessing phase. As mentioned above, replacing flux maximization with another technique, such as an infeasibility test, would also change the time and memory requirements of this portion of the algorithms.

## Conclusion

The WW algorithm consistently achieves significant performance improvements over both the MS and SLP algorithms for the microbial networks we examined. For these networks, the execution time and maximum memory consumption of the WW algorithm are both smaller by multiple factors. The scaling behavior of the WW algorithm as a function of network size is also preferable to both the MS and SLP algorithms. Due to combinatorial explosion during extreme pathway identification, however, all of the algorithms fail to identify the cycles within the human metabolic network. At this point in time, the WW algorithm appears to be the best choice for identifying steady state cycles in large, real-world stoichiometric networks, although additional algorithmic innovation is clearly desirable.

## Methods

### Stoichiometric networks

Genome-scale stoichiometry matrices for *H. pylori *[28], *M. barkeri *[29], *E. coli *[30], *S. cerevisiae *[31], and *H. sapiens *[32] were constructed from the files Hpylori_341_model_smbl.zip, Mb_iAF692.xml, E_coli_AF1260.xml, Sc_iND750.xml, and H_sapien_Recon_1.xml, respectively, which we obtained from [33] and [34]. We followed the SLP convention that reversible reactions are broken into forward and backward reactions, which are represented as two distinct columns in the stoichiometry matrix. Exchange reactions were not included in stoichiometry matrices. The matrices thus constructed were used to define the equation *Sv = 0*, where *S *denotes a stoichiometry matrix and *v *denotes a vector of fluxes.

### Implementations

The Systems Biology Research Tool [35] (version 1.3.0) was used to create the sub-networks of the WW and MS algorithms, using the GNU Linear Programming Kit to solve all linear programs for both algorithms. Metatool [36,37] (version 5.0), in combination with MATLAB (version 7.2), was used to execute the SLP algorithm, since it utilizes the most recent techniques for identifying elementary flux modes [22,36]. Metatool currently uses a 32-bit binary file to identify elementary flux modes, resulting in an upper memory limit of 2^32 ^bytes, that is, 4 GB.

### Performance measurements

The time and memory requirements of each algorithm were used as measures of algorithmic performance. All performance measurements were made on a Dell Precision 490 computer equipped with 8 GB of RAM and a 2.33 GHz Intel Xeon processor with Kubuntu 7.10 (AMD64) as the operating system. A bash script was used to execute 10 programs sequentially for each algorithm and network. The time was recorded before each program began and after each algorithm finished execution to determine the total running time. A perl script (memmon) was used to frequently sample the contents of/proc/meminfo to monitor the memory usage during each program execution. Memory monitoring began before each algorithm was executed to establish a baseline, and the maximum memory consumption during algorithm execution was measured relative to this baseline.

## Authors' contributions

Both JW and AW contributed to the algorithm's conception and participated in drafting the manuscript. JW carried out all performance comparisons.

## Appendix

The following is a list of properties of extreme pathways that have been published previously [14,16], and a list of logical deductions we make based on these properties. Each of these properties and deductions is very simple, but taken together, they lead to the principle (Deduction 8) that allows the WW algorithm to be much more efficient than its predecessors.

Property 1. Extreme pathways are composed of the minimum number of reactions needed to function (that is, to have non-zero flux) at steady state [16].

Property 2. Extreme pathways are systematically independent, that is, extreme pathways cannot be composed of other extreme pathways [14,16].

Property 3. Type III extreme pathways contain only internal reactions, that is, they never contain exchange reactions. All other types of extreme pathways contain at least one exchange reaction [14].

Deduction 1. Given Property 1, if any reaction is removed from an extreme pathway, or inactivated by constraining its flux to zero, the entire pathway is rendered inactive, that is, the flux of each reaction in the pathway must be zero.

Deduction 2. Given Property 1, an extreme pathway can be viewed as a single, independent functional unit. For example, if all reactions in a network are inactivated, except those participating in a given extreme pathway, that particular pathway can still have non-zero flux.

Deduction 3. Given Property 1, a single unidirectional internal reaction can never be an extreme pathway, because it cannot function at steady state by itself (excluding 'null' reactions, like A → A).

Deduction 4. A pair of unidirectional forward-reverse reactions can form a type III extreme pathway. The pair can function at steady state without the need for other reactions and only by functioning together, satisfying Property 1. The pair cannot be composed of other extreme pathways, since a single reaction can never be an extreme pathway (Deduction 3), satisfying Property 2.

Deduction 5. If an extreme pathway is composed of only two unidirectional reactions, they must be a forward-reverse reaction pair (R_F_, R_R_). The only way a reaction pair can maintain steady state concentrations is if R_R _consumes the products of R_F _at the same rate that R_F _consumes the products of R_R_.

Deduction 6. Given Properties 1 and 2, and Deduction 4, an extreme pathway composed of more than two reactions can never contain a forward-reverse reaction pair.

Deduction 7. Given Property 3 and Deduction 2, if the exchange reactions in a stoichiometric network are removed, only type III extreme pathways will remain. (Note, however, that the set of type III pathways in a network may change as a result of exchange reaction removal.)

Deduction 8. If a unidirectional internal reaction R is active (i.e. its flux is greater than zero), all reverse reactions of R are inactivated (i.e. their fluxes have been constrained to zero), and the network contains no exchange reactions, R necessarily participates in a type III extreme pathway composed of more than two reactions. This statement is a direct consequence of Deductions 1, 4, 6 and 7.

## Supplementary Material

Additional file 1 Supplementary Figures. Algorithmic performances represented on a log scale. Click here for file

## References

[B1] Price ND, Reed JL, Palsson BO (2004). Genome-scale models of microbial cells: evaluating the consequences of constraints. Nature Reviews Microbiology.

[B2] Beard DA, Liang S-d, Qian H (2002). Energy balance for analysis of complex metabolic networks. Biophys J.

[B3] Price ND, Famili I, Beard DA, Palsson BO (2002). Extreme pathways and Kirchhoff's second law. Biophys J.

[B4] Price ND, Thiele I, Palsson BO (2006). Candidate states of *Helicobacter pylori *'s genome-scale metabolic network upon application of "loop law" thermodynamic constraints. Biophys J.

[B5] Kümmel A, Panke S, Heinemann M (2006). Systematic assignment of thermodynamic constraints in metabolic network models. BMC Bioinformatics.

[B6] Beard DA, Babson E, Curtis E, Qian H (2004). Thermodynamic constraints for biochemical networks. J Theor Biol.

[B7] Yang F, Qian H, Beard DA (2005). Ab initio prediction of thermodynamically feasible reaction directions from biochemical network stoichiometry. Metab Eng.

[B8] Nigam R, Liang S (2005). A pivoting algorithm for metabolic networks in the presence of thermodynamic constraints. Proc IEEE Comput Syst Bioinform Conf.

[B9] Qian H, Beard DA (2006). Metabolic futile cycles and their functions: a systems analysis of energy and control. Syst Biol (Stevenage).

[B10] Papin JA, Palsson BO (2004). The JAK-STAT signaling network in the human B-cell: an extreme signaling pathway analysis. Biophys J.

[B11] Papin JA, Palsson BO (2004). Topological analysis of mass-balanced signaling networks: a framework to obtain network properties including crosstalk. J Theor Biol.

[B12] Ferrell JE (2002). Self-perpetuating states in signal transduction: positive feedback, double-negative feedback and bistability. Curr Opin Cell Biol.

[B13] Freeman M (2000). Feedback control of intercellular signalling in development. Nature.

[B14] Schilling CH, Letscher D, Palsson BO (2000). Theory for the systemic definition of metabolic pathways and their use in interpreting metabolic function from a pathway-oriented perspective. Journal of Theoretical Biology.

[B15] Schuster S, Fell DA, Dandekar T (2000). A general definition of metabolic pathways useful for systematic organization and analysis of complex metabolic networks. Nat Biotechnol.

[B16] Papin JA, Stelling J, Price ND, Klamt S, Schuster S, Palsson BO (2004). Comparison of network-based pathway analysis methods. Trends Biotechnol.

[B17] Wagner C, Urbanczik R (2005). The Geometry of the Flux Cone of a Metabolic Network. Biophysical Journal.

[B18] Gagneur J, Klamt S (2004). Computation of elementary modes: a unifying framework and the new binary approach. BMC Bioinformatics.

[B19] Terzer M, Stelling J (2006). Accelerating the computation of elementary modes using pattern trees. Alg in Bioinformatics.

[B20] Papin JA, Price ND, Wiback SJ, Fell DA, Palsson BO (2003). Metabolic pathways in the post-genome era. Trends Biochem Sci.

[B21] Urbanczik R, Wagner C (2005). An improved algorithm for stoichiometric network analysis: theory and applications. Bioinformatics.

[B22] Klamt S, Gagneur J, von Kamp A (2005). Algorithmic approaches for computing elementary modes in large biochemical reaction networks. Syst Biol (Stevenage).

[B23] Bell SL, Palsson BO (2005). expa: a program for calculating extreme pathways in biochemical reaction networks. Bioinformatics.

[B24] Mahadevan R, Schilling CH (2003). The effects of alternate optimal solutions in constraint-based genome-scale metabolic models. Metab Eng.

[B25] Motzkin TS, Raiffa H, Thompson GL, Thrall RM (1953). The double description method. Contributions to the Theory of Games.

[B26] Avis D (1998). Computational experience with the reverse search vertex enumeration algorithm. Optimization Methods and Software.

[B27] Avis D A revised implementation of the reverse search vertex enumeration algorithm. Polytopes-Combinatorics and Computation.

[B28] Thiele I, Vo TD, Price ND, Palsson BO (2005). Expanded metabolic reconstruction of *Helicobacter pylori *(iIT341 GSM/GPR): an in silico genome-scale characterization of single- and double-deletion mutants. J Bacteriol.

[B29] Feist AM, Scholten JCM, Palsson BO, Brockman FJ, Ideker T (2006). Modeling methanogenesis with a genome-scale metabolic reconstruction of *Methanosarcina barkeri*. Mol Syst Biol.

[B30] Feist AM, Henry CS, Reed JL, Krummenacker M, Joyce AR, Karp PD, Broadbelt LJ, Hatzimanikatis V, Palsson B (2007). A genome-scale metabolic reconstruction for *Escherichia coli* K-12 MG1655 that accounts for 1260 ORFs and thermodynamic information. Molecular Systems Biology.

[B31] Duarte NC, Herrgard MJ, Palsson BO (2004). Reconstruction and validation of *Saccharomyces cerevisiae *iND750, a fully compartmentalized genome-scale metabolic model. Genome Research.

[B32] Duarte NC, Becker SA, Jamshidi N, Thiele I, Mo ML, Vo TD, Srivas R, Palsson BO (2007). Global reconstruction of the human metabolic network based on genomic and bibliomic data. Proc Natl Acad Sci U S A.

[B33] Systems Biology Research Group. http://gcrg.ucsd.edu.

[B34] BiGG Database. http://bigg.ucsd.edu.

[B35] Wright J, Wagner A (2008). The Systems Biology Research Tool: evolvable open-source software. BMC Systems Biology.

[B36] Kamp A, Schuster S (2006). Metatool 5.0: fast and flexible elementary modes analysis. Bioinformatics.

[B37] Pfeiffer T, Sanchez-Valdenebro I, Nuno JC, Montero F, Schuster S (1999). METATOOL: for studying metabolic networks. Bioinformatics.

