# Supplementary material for: Exhaustive identification of steady state cycles in large stoichiometric networks
Source: BMC Syst Biol. 2008 Jul 11;2:61. doi: 10.1186/1752-0509-2-61 (PMC2478680; doi:10.1186/1752-0509-2-61)
Supplement: Additional file 1 — Supplementary Figures. Algorithmic performances represented on a log scale. [file 1752-0509-2-61-S1.ppt]

## Slide 1
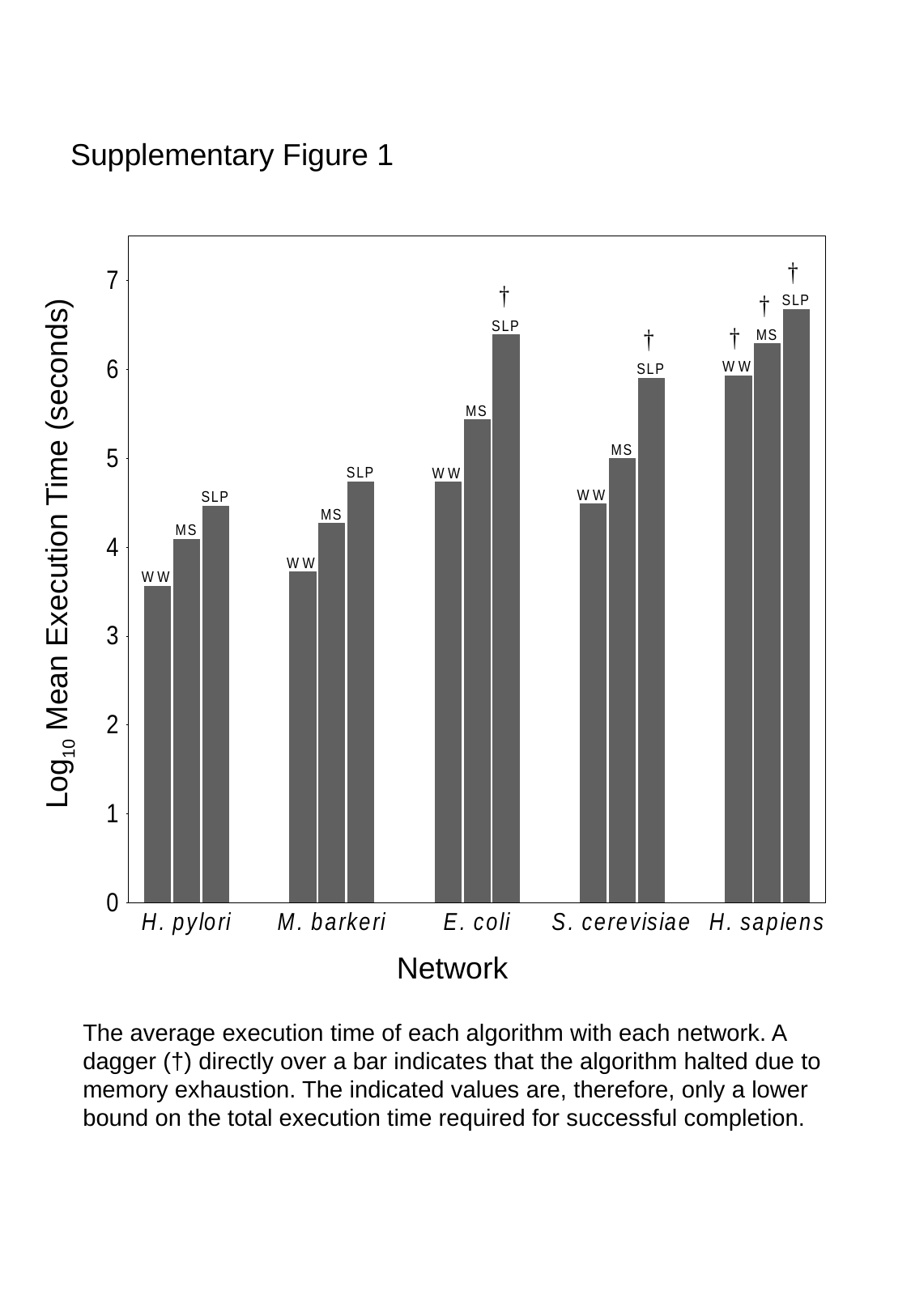

Supplementary Figure 1
Log10 Mean Execution Time (seconds)
Network
The average execution time of each algorithm with each network. A dagger (†) directly over a bar indicates that the algorithm halted due to memory exhaustion. The indicated values are, therefore, only a lower bound on the total execution time required for successful completion.

## Slide 2
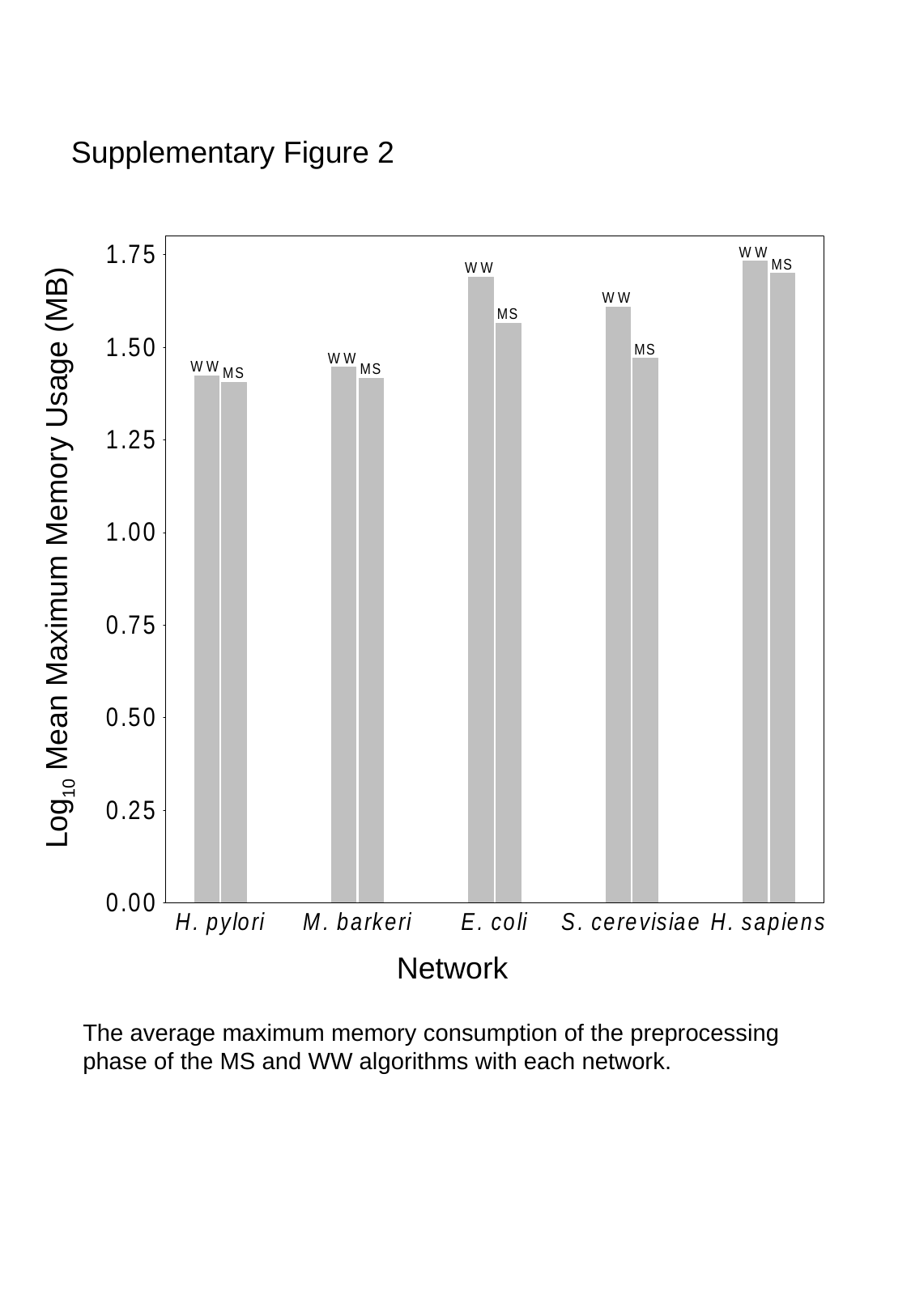

Supplementary Figure 2
Log10 Mean Maximum Memory Usage (MB)
Network
The average maximum memory consumption of the preprocessing phase of the MS and WW algorithms with each network.

## Slide 3
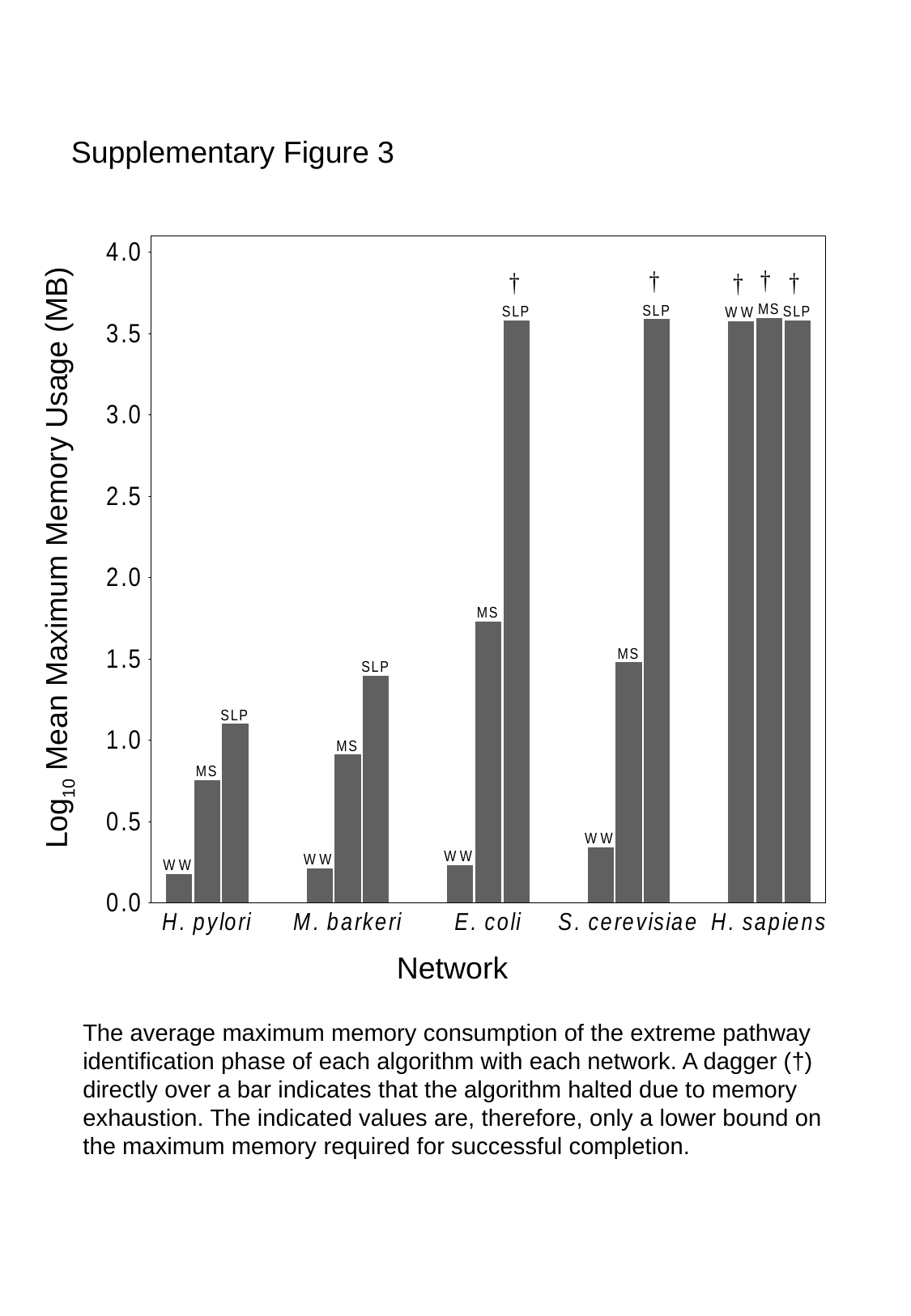

Supplementary Figure 3
Log10 Mean Maximum Memory Usage (MB)
Network
The average maximum memory consumption of the extreme pathway identification phase of each algorithm with each network. A dagger (†) directly over a bar indicates that the algorithm halted due to memory exhaustion. The indicated values are, therefore, only a lower bound on the maximum memory required for successful completion.
